# Sleeping arrangement and house structure affect bed net use in villages along Lake Victoria

**DOI:** 10.1186/1475-2875-9-176

**Published:** 2010-06-22

**Authors:** Hanako Iwashita, Gabriel Dida, Kyoko Futami, George Sonye, Satoshi Kaneko, Masahiro Horio, Hitoshi Kawada, Yoshihide Maekawa, Yoshiki Aoki, Noboru Minakawa

**Affiliations:** 1 Graduate School of International Health Development, Nagasaki University, 1-12-4 Sakamoto, Nagasaki, Nagasakki 852-8523, Japan; 2 School of Public Health, Maseno University, Maseno, Kenya; 3 Institute of Tropical Medicine (NEKKEN) and the Global Center of Excellence Program, Nagasaki University, 1-12-4 Sakamoto, Nagasaki, Nagasaki 852-8523, Japan; 4 Springs of Hope, Mbita, Kenya

## Abstract

**Background:**

Although insecticide-treated bed nets are effective tools, use often does not follow ownership. House structure and space arrangements may make the attempt to use bed nets difficult, especially for school age children. The objectives of this study were to explore whether an individual's sleeping arrangements and house structure affect bed net use in villages along Lake Victoria in western Kenya.

**Methods:**

Sleeping arrangements of residents were directly observed for use of a bed net, use of a bed, and location. House size, number and types of rooms, bed availability, and residents' ages were estimated. The family heads and mothers were asked about the reason for not using bed nets. Individual bed net use was examined against age and sleeping arrangement. Net use at the household level was examined against four variables: bed availability, bed net availability, house size, and number of rooms.

**Results:**

Bed net use by children between five and 15 years of age was lower than that among the other age classes. However, age was dropped from the final model, and sleeping arrangement was significantly associated with net use. Net use was significantly associated with bed availability, number of rooms and their interaction.

**Conclusion:**

Net use was affected by sleeping arrangement and availability of suitable locations for hanging nets, in addition to net availability. Most residents had likely not realized that sleeping arrangement was a factor in net use. The ease of hanging a net is particularly important for children.

## Background

The burden of malaria is a major threat to developing countries, especially in sub-Saharan Africa. Studies showed that insecticide-treated bed nets (ITNs) are effective tools in the prevention of malaria. The use of ITNs alone significantly reduces morbidity and mortality due to malaria. In western Kenya, where intense perennial malaria transmission occurs, a randomized controlled trial confirmed that ITNs reduce all-cause child mortality in children aged less than five years by 16% [1]. ITN use was associated with a 44% reduction in mortality in Kenya [2]. This level of protection corresponds to about seven deaths averted for every 1000 ITNs distributed [2].

In 1998, the WHO launched the Roll Back Malaria movement. One of its primary objective is to increase ITN coverage among vulnerable groups to over 60%. The WHO later revised this ITN objective to reach 80% coverage by 2015 [3]. Under the new malaria strategy, high coverage of ITNs for vulnerable groups, particularly young children and pregnant women is the cornerstone of malaria prevention [4]. Recently, in addition to the conventional ITNs, the WHO Pesticide Testing Scheme recommends long-lasting insecticidal nets (LLINs) [5]. Insecticides are applied in LLINs in a factory, and they last for several years without requiring pesticide reapplication.

Large increases in funding and attention to malaria have recently accelerated malaria control activities [3]. Rapid scaling up of ITNs coverage was reported in many African countries [6]. In 2006, the National Malaria Control Programmes distributed 7.1 million ITNs [3]. In Kenya, ITNs have been primarily distributed to pregnant women and children under five years of age, either free of charge or at subsidized prices, by the Kenya Ministry of Health and nongovernmental organization (NGOs). However some bed nets studies show that the incidence of use does not follow that of ownership [7-10]. A study in western Kenya found that 30% of bed net recipients did not adhere to net use [9]. Their attempt to use bed nets might be hampered by environmental, social and cultural reasons.

Some studies reported that children under five years of age and pregnant women are more likely to use a bed net than children between five to 15 years of age [11-15]. Distribution of ITNs have been targeted to these age groups, because they are most vulnerable to malaria [3]. Consequently older children, mainly school-aged children are least protected by ITNs. However, other age groups also should be protected. Children older than five years and adults are also vulnerable to severe illness, death, and substantial economic costs from the disease. In addition, they can become a reservoir of *Plasmodium falciparum *[16]. Use of ITNs can extend protection to the surrounding community, providing protective effects beyond the individual. To have maximum effect within communities, ITN coverage should be as high as possible throughout all age groups [16].

In African countries, infants share the same bed space with their mothers to facilitate breast feeding[17]. However, older children sleep mainly in the living rooms without their mothers [17]. In living rooms, bed nets have to be removed and re-hung frequently. The inconvenience of frequent mountings is a problem for children, in particular [17,18]. It has been suggested that house structure and space arrangement make the problem more difficult [19]. However, this notion was mainly based on casual observations and stories from residents, and has not been studied in detail. This study explored the effects of sleeping arrangement and house structure on bed net use in villages along Lake Victoria in western Kenya.

## Methods

### Study area

The study area (4.4 km^2^) included three villages (Koguna, Nyachabe, and Tabla) along Lake Victoria in Mbita district, Western Kenya. The rainfall pattern in the area is bimodal, with the long rainy season occurring from March through May, and the short rainy season in November and December. Intense malaria transmission occurs throughout the year. Three species of vectors were known: *Anopheles arabiensis, Anopheles gambiae*, and *Anopheles funestus *[20].

The area had 455 households. Most houses are constructed of a stick framework plastered with a mixture of mud and cow dung, and a corrugated iron roof. A few houses have thatched roofs. The majority of residents belong to the Luo ethnic group. Although Luo is the main language spoken, many also speak English and Swahili. The major income sources are fishing and traditional small-scale farming. Some residents are temporary migrants who depend on fishing.

### Data collection

One hundred houses were chosen randomly (31 houses in Koguna, 39 in Nyachabe and 25 in Tabla), using the latest data set obtained by the demographic surveillance system (DSS). Nagasaki University, Japan, and Kenya Medical Research Institute, Kenya, deployed the DSS in 2007. The houses were surveyed on six separate dates early in May 2009 (during the rainy season). During the early morning hours (4:30-6:00 hours), each house was visited jointly with a team that was sampling mosquitoes for a separate study. Residents in these houses paid attention to collecting mosquitoes and their usual usage of bed net was easily monitored. In the course of sampling mosquitoes, the sleeping arrangements of the residents were directly observed for (1) use of a bed net, (2) use of a bed, and (3) location. The ages of the residents were estimated and categorized in three classes: (1) under five years of age (infants), (2) between five and 15 years of age (children), and (3) over 15 years (adults). Sleeping arrangements of those residents who opened doors or had already left their sleeping sites were confirmed through an interview. Three local assistants were employed to undertake this task.

The team sampling the mosquitoes informed the residents in advance of the dates of its visit. However, the residents were not informed of the dates of the bed net survey when they signed the consent form. This was to avoid any possible effects on bed net use by informing residents in advance. The procedure design was based on the method used in the study of Alaii *et al *[9].

A few hours after the morning survey, the houses were revisited. Family heads or mothers were interviewed to confirm sleepers' ages and sleeping arrangements, as recorded in the morning observation. If the interviewees' descriptions differed from those of the earlier survey, the information was verified by interviewing other family members. The bed nets hung during the daytime visit were counted, and their locations were noted. Residents were also asked about the number of nets not being used. Bed nets were categorized as LLINs and non-LLINs. The floor area (m^2^) of each house was measured using a tape measure. The open floor area without furniture, and the sizes of the beds were also measured, and both measurements were used to estimate a potential area for hanging bed nets in each house. The potential area was divided by one bed net size (m^2^) to estimate the potential number of bed nets that could be hung in each house. If any house included residents who had slept without nets the previous night, the family heads or mothers of the houses were asked about the reasons for this. They were also asked about the purposes of using nets.

### Ethical consideration

This study was approved by the Ethical Committee of Graduate School of International Health Development, Nagasaki University, Kenya Medical Research Institute (KEMRI) and Maseno University, Kenya. Informed consent was obtained from all heads of households after the study was explained in the local language.

### Data analyses

A generalized linear mixed model (GLMM) using the binomial distribution was used to examine whether residents' ages and sleeping arrangements explained bed net use using the package lme4 in R [21]. Village was also included in the analysis as an explanatory variable (categorical), because bed net use might be different from one village to another. Bed net use was a binary response variable (slept with or without a net). Age was an explanatory variable categorized into three classes. Sleeping arrangement was categorized by four types: (1) slept on a bed in a bedroom, (2) slept on a bed in a non-bedroom, (3) did not sleep on a bed in a bedroom, and (4) did not sleep on a bed in a non-bedroom. Non-bedrooms included living rooms and combination rooms. A combination room was one used for a dual purpose in a house with only one room. When a mattress was used without a bed frame, and when a resident slept on a sofa, these sleeping arrangements were considered "not slept on a bed." Since most houses had multiple individuals, house was considered as a random intercept. A backward selection procedure was applied for modelling, and the selection was based on the log likelihood ratio test [22].

The same modeling procedure was used to explore whether any locations were more suitable for hanging nets, as the availability of suitable locations may affect bed net use. The response variable (binary) was whether a net used during the previous night was also hung or taken down in the course of the following day. The sleeping arrangements in the above modelling were used as probable locations for hanging. Some nets are taken down every morning, and hung or used the following night as necessary. The other nets hung throughout the day were more likely to remain at the same locations for a while, and the residents who sleep at the locations with nets likely use those nets because they are readily available. That is, the locations where nets were hung during the day were considered suitable locations. A GLMM was used to verify whether the nets hung during the day were more likely used during the night. Village was also included in the analysis as an explanatory variable.

A GLMM with binomial distribution was also used to examine the relationships of four variables, net use with (1) bed availability, (2) bed net availability, (3) house size, (4) number of rooms, and (5) village. Bed net use was defined as the ratio of the number of residents who slept with nets to the number of those without nets in a house. The variable of bed availability was total bed area (m^2^) in a house, and house size was total floor area (m^2^). The values for these variables, excluding the number of rooms, were divided by the adjusted number of residents in each house. The adjusted number of residents was estimated based on body size: an infant was treated as 0.3 person, a child as 0.5 person, and an adult as one person. If the number of rooms in a house had been divided by the number of residents, the qualitative characteristics of the rooms that the data captured might have been lost. For example, in a house with two or three rooms, one was used as a living room, and the others were usually used as bedrooms. Using the raw number of rooms maintained both this characteristic and the quantitative characteristics. Sampling dates were considered as a random effect, and the modeling allowed for a random slope to find optimal random errors [23]. Collinearity between the variables was assessed using graphs, correlation coefficients, and variance inflation factors (VIF).

The effects of bed availability, house size, and the number of rooms may be more profound in the case of houses that had enough bed nets, because the effects of net availability did not then exist, which excluded the age effect from the selective distribution to infants and pregnant women. In a separate GLMM, the data set from houses with enough nets was used to examine whether these variables were associated with net use. When a house had more than one net per two residents, the house was considered to have enough nets. This criterion was used because the Kenya Malaria Monitoring and Evaluation Plan had set this as a goal [24].

## Results

Of the 100 selected houses, 95 were surveyed. Five large concrete houses were excluded from the survey because concrete houses do not represent the ordinary houses in the area. The mean size of houses was 16.7 (SD = 6.0) m^2^. The mean numbers of rooms and beds were 1.7 (SD = 0.8) and 0.9 (SD = 0.6) per house, respectively. In total, 388 residents and 289 nets were counted, and their means were 4.1 (SD = 1.9) and 3.0 (SD = 1.2) per house, respectively. Of the 289 nets, 132 (45.7%) nets were used, and 309 (79.6%) residents slept under nets. Over 80% (52 nets) of them were LLINs. The residents obtained the nets mainly from health centres, NGOs, shops or schools. None slept outside. That is, 0.7 nets were available per resident, and 2.3 residents slept per net. The mean potential number of bed nets that could be hung was 2.6 (SD = 1.2) per house.

A total of 86 family heads or mothers provided reasons for using nets. The most popular reason (n = 79) for using nets was to prevent mosquito bites (Table 1). Family heads or mothers of 25 houses that had non-bed net users were asked about not using nets. Nine of them could not provide a proper answer. The most frequent reason given (n = 5) was that there were no mosquitoes.

**Table 1:** Reasons for using and not using bed nets

	Number	%
Reasons for using bed nets		
To prevent mosquitoes biting	79	91.9
To prevent getting malaria	7	8.1
Number of interviewees	86	100.0
Reasons for not using bed nets		
There are no mosquitoes	5	20.0
There are no extra nets (but some one has an extra one)	3	12.0
Net is old	3	12.0
Net is new	1	4.0
We lost it	1	4.0
We use it for storing millet	1	4.0
It is hot	1	4.0
There is a sick person	1	4.0
No reason	9	36.0
Number of interviewees	25	100.0

Bed net use by children was lower than that among the other age classes (Table 2). However, when the explanatory variables, age, sleeping arrangement, and village were examined against an individual's bed net use, age, village and the interaction terms were dropped from the final model. Sleeping arrangement was statistically significant in relation to net use (*n *= 388, *df *= 3, deviance = 96.45, *p *< 0.001). The net use of residents who slept on beds in bedrooms was greater than that in the other arrangements. The net use of residents on beds in non-bedrooms was greater than that of those without beds in non-bedrooms. Sleeping arrangement differed significantly among the age classes (*n *= 388, *df *= 6, chi-square = 100.13, *p *< 0.001). Nearly 80% of children slept without beds in non-bedrooms, and only 10% of them slept in bedrooms (Table 3). The sleeping arrangements of infants and adults were similar, and over 50% of them slept in bedrooms.

**Table 2:** Bed net use among different age classes and sleeping arrangements

Variables	With net	Without net	Total
Age			
Infant (<5 years)	140 (87.0) ^†^	21 (13.0)	161 (41.5)
Child (5-15 years)	64 (66.0)	33 (34.0)	97 (25.0)
Adult (above 15 years)	105 (80.8)	25 (19.2)	130 (33.5)
Total	309 (79.6)	79 (20.4)	388
Sleeping arrangement			
Bed in bedroom	97 (97.0)	3 (3.0)	100 (25.8)
Bed in non-bedroom	84 (88.4)	11 (11.6)	95 (24.5)
Non-bed in bedroom	18 (78.3)	5 (21.7)	23 (5.9)
Non-bed in non-bedroom	110 (64.7)	60 (35.3)	170 (43.8)
Total	309 (79.6)	79 (20.4)	388

^†^Values in prentices are percentages.

**Table 3:** Differences in sleeping arrangements among infants, children, and adults

Locations	Infant	Child	Adult	Total
Bed in bedroom	61 (37.9) ^†^	6 (6.2)	33 (25.4)	100 (25.8)
Bed in non-bedroom	59 (36.6)	4 (4.1)	32 (24.6)	95 (24.5)
Non-bed in bedroom	5 (3.1)	11 (11.3)	7 (5.4)	23 (5.9)
Non-bed in non-bedroom	36 (22.4)	76 (78.4)	58 (44.6)	170 (43.8)
Total	161 (41.5)	97 (25.0)	130 (33.5)	388

^†^Values in parentheses are percentages.

In the analysis of suitable location, the response variable whether nets were hung or taken down during the day was significantly associated with probable locations for hanging (*n *= 132, *df *= 3, deviance = 41.11, *p *< 0.001), but village was dropped from the final model. More nets were hung on the beds in bedrooms during the day than in the other locations, and beds in non-bedrooms had more nets hung than non-bed sites in non-bedrooms (Table 4). Of 103 nets hung during the day, 94 (91.3%) nets were used during the night, and the association was statistically significant (*n *= 289, *df *= 1, chi-square = 142.20, *p *< 0.001).

**Table 4:** Locations of bed nets hung at night and taken down during the day

Locations	Hung	Taken down	Total
Bed in bedroom	44 (97.8) ^†^	1 (2.2)	45 (34.1)
Bed in non-bedroom	26 (78.8)	7 (21.2)	33 (25.0)
Non-bed in bedroom	5 (55.6)	4 (44.4)	9 (6.8)
Non-bed in non-bedroom	19 (42.2)	26 (57.8)	45 (34.1)
Total	94 (71.2)	38 (28.8)	132

^†^Values in parentheses are percentages.

Net use was examined using five variables, bed availability, bed net availability, house size, number of rooms, and village. An assessment of multicollinearity indicated that house size was highly correlated with net availability (*r *= 0.76). The VIF of house size was 2.8 and, therefore, house size was excluded from the modelling. The other reason for excluding this variable was that there was a greater interest in the relative importance of net availability. Bed availability, bed net availability and number of rooms remained in the final model, but village was dropped from the final model. Net availability, the number of rooms, and the interaction between bed availability and the number of rooms were statistically significant (Table 5). However, the variable of bed availability was not statistically significant. The coefficients suggest that the number of rooms and interactions were relatively more important than bed availability. Eighty (84.2%) houses had enough bed nets to cover all house residents. When the houses with insufficient nets were excluded from the modeling, bed availability, the number of rooms and their interaction remained in the final model (Table 5).

**Table 5:** Results from the generalized linear mixed models examining the associations of bed net use with bed availability, net availability, number of rooms, and their interactions, using the data from all houses, and those from houses with enough bed nets

Variables in the final model	Coefficient	Standard error	*z*-value	*p*-value
All houses (*n *= 95)				
Bed availability (m^2^/person)	3.90	2.25	1.73	0.083
Net availability (m^2^/person)	0.78	0.31	2.52	0.011*
Number of rooms	1.32	0.52	2.55	0.011*
Bed availability × number of rooms	-1.23	0.57	-2.16	0.031*
Houses with enough nets (*n *= 80)				
Bed availability	10.31	4.37	2.36	0.018*
Number of rooms	3.32	0.96	3.47	<0.001*
Bed availability × number of rooms	-2.85	0.85	-3.36	<0.001*

Bed availability was included in the first model as a random variable.

*Statistically significant at *p *< 0.05 level.

## Discussion

Although bed net use differed among the age classes, removing the age variable from the model and using sleeping arrangement alone resulted in better prediction. In the study area, bed net use by children was less frequent than that of infants and adults. Infants usually sleep with their parents, and the usage patterns of these two age classes were similar. This finding is comparable to those of other studies [11-15]. Other studies claim that the difference in use is due to a strategy for distributing nets that prioritizes infants and their mothers. However, the results from this study suggest that the lower use by children is due to sleeping arrangements. More children slept without a bed in living rooms and combination rooms because infants with parents were given priority in using beds in bedrooms. Since nearly 85% of houses had enough nets to cover all residents, the low use by children was unlikely to have been due to an insufficiency of nets. Moreover, dropping the age variable suggests that low net use also occurred in the case of infants and adults who slept without beds in bedrooms and/or in the other rooms.

The results from the daytime survey suggest that the most suitable location for hanging nets was over beds in bedrooms. Even in non-bedrooms, nets were hung over nearly 80% of beds. That is, most nets on beds were hung during the day, whereas over 50% of nets in non-bed sites in non-bedrooms were taken down. Nets in non-bed sites probably interfered with the daily activities (e.g., cooking) of humans and of animals such as chickens. In fact, over 40% of houses kept chickens in non-bedrooms, and most non-bedrooms had a sofa set. Residents had to rearrange furniture to create sleeping area in non-bedrooms everyday. Therefore, the nets were more likely to be taken down to increase space for various daily activities in non-bed rooms. This accounts for the lower number of nets hung in non-bedrooms during the day. In fact, the number of nets hung in non-bed sites in bedrooms was greater than that in non-bed sites in the other rooms.

The instability of sleeping sites is another probable reason why more nets were taken down in non-bed sites. Non-bed sleeping sites were usually on an open floor space, and daily activities may force residents (mostly children) to shift their sleeping sites. For example, a guest sleeper reduces the sleeping space for residents in a living room or a combination room. Children must then shift their sleeping sites. Moreover, many children often shift their sleeping houses. Their frequent shifts of sleeping sites may reduce their attachment to bed nets.

Nets are probably more easily hung over a bed using a frame. Bedrooms are usually narrower than the other rooms, and so nets are easily deployed on a bed by tying the strings to nearby objects. Consequently, residents can easily access the nets being hung with no extra effort, and then use in such sites increases.

The variables bed availability, net availability, and number of rooms remained in the final model examining bed net use at household level. The importance of net availability indicates that some houses lacked a sufficient number of nets to cover all residents. Interestingly, the variable of bed availability alone was not statistically significant, despite other analyses that suggest the significance of beds for net use. However, bed availability became significant when it interacted with the number of rooms. The variable of the number of rooms was by itself statistically significant. A study in Burkina Faso reported the relationship between net use and the number of rooms based on a qualitative data set from observation [19]. The present study confirmed the relationship by analyzing a quantitative data set using direct observation. As the number of rooms increased, the role of each room became clearer: when a house had more than two rooms, one room was usually used as a living room, and the others as bedrooms. Consequently, having more bedrooms increases both privacy and the space available for beds, which in turn increases the number of sites that are suitable for hanging nets, thereby increasing net use. The analysis for houses with enough nets also confirmed this notion.

## Conclusion

This study revealed that net use was affected by sleeping arrangement and availability of suitable locations for hanging nets, in addition to net availability. When residents were asked their reasons for not using nets, some responded that there were few mosquitoes, but none mentioned the sleeping arrangements. "Few mosquitoes" is also a popular response from residents in other study areas [8,19]. While the number of mosquitoes may affect use, most residents had probably not realized that sleeping arrangement and availability of suitable locations for nets were factors in net use, as the number of mosquitoes was expected to be high because this study was conducted during the rainy season [20].

### Implications

The numbers of rooms and beds could be increased to improve bed net use. However, such a practice would be unrealistic, because most residents cannot afford it. Moreover, it is difficult to have multiple beds in houses with an average size of 16.7 m^2^. It is advisable to improve the tools for hanging nets. Hanging nets may become easier if girders or poles are installed, to which the strings of a net can be tied. As nearly 70% of residents slept in living rooms and combination rooms, it is practical to introduce methods that enable them to hang a net easily in these rooms. The ease of hanging a net is particularly important for children.

Another implication of this study is that it is important to identify those houses without enough bed nets before nets are distributed. Most houses in the study area had enough bed nets, and in fact, several had more than enough. Despite this, NGOs continued to distribute nets without identifying which houses in the area lacked nets. Some residents asked NGOs or heath centers for extra nets because nets are used for various different purposes, such as storing crops or fishing[25]. However, this study found that some houses still lacked nets, and the distribution of bed nets should target those houses.

## Competing interests

The authors declare that they have no competing interests.

## Authors' contributions

HI and NM conceived and designed the study. HI organized the field work and collected data, with assistance from GD,GS, HK and YM. SK provided data from DSS. NM and MH performed the data analyses. HI drafted the first manuscript, and NM and YA finalized the manuscript. All authors have read and approved the final manuscript.
